# Physical and Biological Evaluation of Low-Molecular-Weight Hyaluronic Acid/Fe_3_O_4_ Nanoparticle for Targeting MCF7 Breast Cancer Cells

**DOI:** 10.3390/polym12051094

**Published:** 2020-05-11

**Authors:** Hsin-Ta Wang, Po-Chien Chou, Ping-Han Wu, Chi-Ming Lee, Kang-Hsin Fan, Wei-Jen Chang, Sheng-Yang Lee, Haw-Ming Huang

**Affiliations:** 1 School of Organic and Polymeric, National Taipei University of Technology, Taipei 10608, Taiwan; htwang@mail.ntut.edu.tw (H.-T.W.); jack19186@hotmail.com (P.-C.C.); 2 School of Dentistry, College of Oral Medicine, Taipei Medical University, Taipei 11031, Taiwan; m8404006@tmu.edu.tw (W.-J.C.); seanlee@tmu.edu.tw (S.-Y.L.); 3 Graduate Institute of Biomedical Materials and Tissue Engineering, College of Biomedical Engineering, Taipei Medical University, Taipei 11031, Taiwan; gahwclbjwph@hotmail.com; 4 Core Facility Center, Office of Research and Development, Taipei Medical Universitry, Taipei 11031, Taiwan; jaunslee@tmu.edu.tw; 5 Dental Department, En Chu Kong Hospital, New Taipei City 23742, Taiwan; dentaddie@yahoo.com.tw; 6 Graduate Institute of Biomedical Optomechatronics, College of Biomedical Engineering, Taipei Medical University, Taipei 11031, Taiwan

**Keywords:** low molecular weight, hyaluronic acid, TOF-SIMS, nanoparticles, iron oxide

## Abstract

Low-molecular-weight hyaluronic acid (LMWHA) was integrated with superparamagnetic Fe_3_O_4_ nanoparticles (Fe_3_O_4_ NPs). The size distribution, zeta potential, viscosity, thermogravimetric and paramagnetic properties of the LMWHA-Fe_3_O_4_ NPs were systematically examined. For cellular experiments, MCF7 breast cancer cell line was carried out. In addition, the cell targeting ability and characteristics of the LMWHA-Fe_3_O_4_ NPs for MCF7 breast cancer cells were analyzed using the thiocyanate method and time-of-flight secondary ion mass spectrometry (TOF-SIMS). The experimental results showed that the LMWHA-Fe_3_O_4_ NPs were not only easily injectable due to their low viscosity, but also exhibited a significant superparamagnetic property. Furthermore, the in vitro assay results showed that the NPs had negligible cytotoxicity and exhibited a good cancer cell targeting ability. Overall, the results therefore suggest that the LMWHA-Fe_3_O_4_ NPs have considerable potential as an injectable agent for enhanced magnetic resonance imaging (MRI) and/or hyperthermia treatment in breast cancer therapy.

## 1. Introduction

In clinical contexts, the sensitivity and diagnosis accuracy of magnetic resonance (MR) images are commonly enhanced by injecting a contrast agent into the body prior to scanning [1]. One of the most commonly used agents is gadolinium (Gd); a ductile rare-earth element with ferromagnetic and paramagnetic properties below and above a temperature of 20 °C, respectively [2]. However, with their good biocompatibility, high sensitivity and ease of obtention [3], magnetic nanoparticles (NPs) have attracted increasing interest for a wide range of biomedical applications nowadays, including hyperthermia therapy, drug delivery, biochemical sensing, and tissue repair [4]. Among the various magnetic nanoparticles available, magnetite (Fe_3_O_4_) exhibits excellent superparamagnetic properties, and is thus increasingly used in place of Gd as a contrast agent for the MR imaging of tumors [5,6,7,8].

Hyaluronic acid (HA) is a biopolymer glycosaminoglycan, which is located mainly in the connective tissue, epidermal tissue, neural tissue and joint tissues in biological organisms [9], and serves as the main component of the extracellular matrix [10]. In medical applications, HA is commonly used in the treatment of osteoarthritis and skin wounds. Furthermore, since it readily binds to the CD44 receptors on tumor cells [10,11], HA is also used as a tumor targeting material [12,13]. HA retains excellent viscoelasticity following water absorption, and is thus useful in retaining skin moisture and treating osteoarthritis [14]. However, with a molecular weight of around 2000 kDa, nature HA has a high viscosity and is hence not easily injected into the human body through the blood vessels. Accordingly, several groups have investigated the physiological functions of low molecular weight hyaluronic acid (LMWHA) (80–800 kDa) [15,16]. It was reported that LMWHA exhibits a potentially beneficial effect on wound healing immune response and angiogenesis [17]. 

Zhang et al. (2014) used HA in combination with magnetic nano-particles as a contrast agent for enhancing the quality of MR tumor images through targeting with the CD44 receptors on the tumor cell surface [18]. Li et al. used polyethyleneimine-stabilized Fe_3_O_4_ NPs to integrate LMWHA with two different molecular weights (6 and 31 kDa) and showed that both LMWHAs served as effective probes for the MR imaging of cancer cells with overexpressed CD44 receptors [19]. Zhong et al. (2019) used LMWHA with molecular weights of 7, 63 and 102 kDa to fabricate LMWHA-NPs for drug release. The results indicated that the HA-NPs targeted the CD44 receptors in a molecular weight dependent manner [20]. 

However, while the aforementioned studies evaluated the targeting ability and performance of LMWHA-NPs using in vitro cellular and in vivo animal tests, the physical properties of the LMWHA-NPs, and in particular, the dynamic viscoelastic properties, were not discussed. Consequently, the synthesis and characterization of LMWHA with a specific molecular weight and a low viscosity for use as an injectable contrast agent for MR imaging remains an important concern. Current techniques for manufacturing LMWHA use two main methods to destroy the main bonds of HMWHA, namely physical methods (e.g., ultrasonic, ozone, electron beam, gamma rays, and heat treatment) and chemical methods (e.g., enzymatic and acid degradation) [21,22,23]. Among these methods, γ-ray irradiation dramatically decreases the dynamic viscosity of the resulting LMWHA under an applied shear rate, and exhibits Newtonian liquid behavior [17,18]. As a result, the feasibility of the LMWHA as an injectable tumor targeting agent is significantly improved. With this regard, the present study also uses a γ-ray technique to fabricate LMWHA. The superparamagnetic Fe_3_O_4_ nanoparticles (Fe_3_O_4_ NPs) was then integrated to LMWHA and the physical and biological characteristics of the resulting LMWHA-Fe_3_O_4_ NPs were systematically examined by X-ray diffraction (XRD), electrophoretic light scattering, viscosity measurements, and superconducting quantum interference device. Finally, the targeting performance of the LMWHA-Fe_3_O_4_ NPs for MCF7 breast cancer cells was analyzed using the thiocyanate method and time-of-flight secondary ion mass spectrometry (TOF-SIMS).

## 2. Materials and Methods 

### 2.1. Materials

FeCl_2_·4H_2_O was purchased from Avantor Performance Materials, Inc. (Allentown, PA, USA). FeCl_3_·6H_2_O, toluene, ammonia solution hydrochloric acid, ammonium persulfate, potassium thiocyanate and oleic acid were purchased from Nacalai Tesque (Kyoto, Japan). Hyaluronic acid (molecular weight 3000 kDa) was purchased from Cheng-Yi Chemical Industry Co. Ltd. (Taipei, Taiwan). All other analytical grade reagents and solvents used were purchased from J.T. Baker (Phillipsburg, NJ, USA). αMEM, L-glutamine, fetal bovine serum, and penicillin-streptomycin were obtained from Gibco (Grand Island, NY, USA). Sodium nitrate was purchased from Merck (KGaA, Darmstadt, Germany). Finally, dimethyl sulfoxide, Triton X-100 and 3-(4,5-Dimethylthiazol-2-yl)-2,5-diphenyltetrazolium bromide (MTT) were obtained from Sigma-Aldrich (St. Louis, MO, USA).

### 2.2. Preparation of Fe_3_O_4_ Nanoparticle

Oleic acid-coated Fe_3_O_4_ nanoparticles were fabricated using the co-precipitation method described in [24]. Briefly, FeCl_2_·4H_2_O and FeCl_3_·6H_2_O were mixed in a ratio of 1:3 and dissolved in degassed distilled water (DD water). After a stirring process, the sample was heated to 85 °C and NH_4_OH was added to achieve a final FeCl_2_·4H_2_O, FeCl_3_·6H_2_O, NH_4_OH, water ratio of 1:2.5:2.5:60. As the sample cooled to room temperature, Fe_3_O_4_ NPs were formed in accordance with the following reaction:

Fe^2+^ + 2Fe^3+^ + 8OH^−^ → Fe_3_O_4_ + 4H_2_O

Oleic acid was added to the prepared NPs at 85 °C under stirring for 30 min. A strong magnet was then used to separate the oleic-coated Fe_3_O_4_ NPs from the solution. The collected NPs were washed in DD water and then dried in an oven at 40 °C for 24 h. The morphology of the coated Fe_3_O_4_ NPs were observed by a transmission electron microscope (TEM, H-600, Hitachi, Ltd., Tokyo, Japan). In addition, the particle size distribution and zeta potential of the NPs were determined using an electrophoretic light scattering device (NanoBrook 90Plus Zeta, Brookhaven Instruments, New York, NY, USA). The measurement results were obtained for samples with a concentration of 0.25 mg/mL and the measurement process was repeated five times for each sample to ensure the reliability of the results. Finally, the structure and crystalline properties of the fabricated Fe_3_O_4_ nanoparticles were examined using an X-ray diffractometer (D/MAX 2000 PC, Rigaku Co., Tokyo, Japan) for incidence angles in the range of 2θ = 20° to 90°.

### 2.3. Preparation of LMWHA-Fe_3_O_4_ NPs

In accordance with the method described in [17], LMWHA was produced by irradiating the purchased HMWHA with a cobalt-60 irradiator (Point Source, AECL, IR-79, Nordion, Ottawa, ON, Canada). The irradiating condition was set at 22 °C for 20 h with a dose rate of 1 kGy/h. The molecular weight of the produced LMWHA was measured by gel permeation chromatography (GPC). Briefly, LMWHA was added to 0.1 M NaCl to form a 10 mg/mL LMWHA solution. 200 μL of the LMWHA solution was injected into a separation device (Series 200, Perkin Elmer, Waltham, MA, USA) equipped with a chromatography column (SB-806M HQ, Shodex, Kanagawa, Japan) at 25 °C. The flow rate of the mobile phase (0.1 M HPLC grade sodium nitrate) in the column was set to 0.5 mL/min. The GPC signals were collected using a refractive index (RI) detector (Series 200, Perkin Elmer, Waltham, MA, USA) and the calibration curve was obtained using a standard kit (Pullulan ReadyCal Kits, PSS Polymer Standards Service, Mainz, Germany). The molecular weight of the LMWHA sample was then determined using commercial ChromManager 5.8 software (ABDC WorkShop, Taichung, Taiwan).

Using the method described in [25], LMWHA-Fe_3_O_4_ NPs were prepared by dissolving 23 mg oleic acid-coated Fe_3_O_4_ NPs in 15 mL of toluene. LMWHA aqueous solution was additionally prepared by adding 25 mg LMWHA to 30 mL NaOH (1 mol/L) solution. The two solutions were placed in a reaction bottle and vigorously stirred for 24 h to replace the hydrophobic oleic acid on the Fe_3_O_4_ NP surface with hydrophilic LMWHA. After continuous rapid stirring for 24 h, the sample was left to stand for 20 min to allow the solution to separate. The light-colored liquid at the bottom of the solution was subjected to ultra-filtration and was then collected by centrifugation at 8000 rpm for 10 min. The pH of the solution was adjusted to 7 through the addition of 0.1 mol/L HCl solution and the excess water was then removed using a freeze dryer.

### 2.4. Determination of Ferrous Ion Content in Fabricated LMWHA-Fe_3_O_4_ NPs

The iron ion content in the fabricated LMWHA-Fe_3_O_4_ NPs was determined by reacting the iron ions with thiocyanate ions in a moderately acidic medium to form a dark red iron thiocyanate complex. In particular, 30% hydrochloric acid was added to the LMWHA-Fe_3_O_4_ NP samples at 55 °C for 3 h followed by the addition of ammonium persulfate for 15 min to form Fe^3+^ ions. Potassium thiocyanate (KSCN) was then added to the solution. The SCN^−^ ions reacted with the Fe^3+^ ions to form a blood-red colored complex in accordance with the following formula:

Fe^3+^ 6SCN^−^ → [Fe(SCN)_6_]^3−^

The color intensity of the complex was determined by a microplate reader (EZ Read 400, Biochrom, Holliston, MA, USA) at a wavelength of 570 nm. The concentration of the iron oxide NPs in the prepared samples was then determined by comparing the measured color intensity with the intensity readings obtained for a series of standard solutions with known Fe^3+^ concentrations.

### 2.5. Characterization of Fabricated LMWHA-Fe_3_O_4_ NPs

The superparamagnetic properties of the fabricated LMWHA-Fe_3_O_4_ NPs were determined using a superconducting quantum interference device (SQUID) (MPMS7, Quantum Design, San Diego, CA, USA). The hysteresis loops of the LMWHA-Fe_3_O_4_ NPs were measured at temperatures of 5 K and 300 K. The saturation magnetizations of the oleic acid-coated Fe_3_O_4_ NPs and LMWHA-Fe_3_O_4_ NPs were also tested and compared. 

The dynamic viscosities of the LMWHA and LMWHA-Fe_3_O_4_ NPs were measured at 25 °C using a viscometer (X-420, Cannon Instrument Co., State College, PA, USA). As described in a previous study [26], the samples were added to pure water to form solutions with a concentration of 0.5 mg/mL. The solutions were then stirred magnetically for 2 h and the dynamic viscosity was read with units of centistokes (cSt) using DD water as a control. 

The thermal stabilities of the oleic acid-coated Fe_3_O_4_ NPs, neat LMWHA, and LMWHA-Fe_3_O_4_ NPs were measured using a thermogravimeter (TGA, TG 209 F3 Tarsus, Netzsch, Gerätebau GmbH, Bavarian, Germany). An amount of 5 mg of each sample was heated from room temperature to 700 °C at a rate of 10 °C/min in a chamber filled with nitrogen. The decomposition temperatures (T_d_) and residual weights of the various samples at 700 °C were then measured and compared.

### 2.6. In Vitro Biocompatibility Tests of LMWHA-Fe_3_O_4_ NPs

MCF7 breast cancer cells were seeded onto Petri dishes with a density of 1 × 10^4^ cells/mL and maintained in alpha modified Eagle’s minimum essential medium (αMEM) supplemented with 4 mM L-glutamine, 10% fetal bovine serum, and 1% penicillin-streptomycin. The cells were incubated at 37 °C in a 5% CO_2_ environment for periods of 3, 6, 9 and 12 h, respectively. The cytotoxicity of the prepared materials was evaluated in accordance with the ISO10993-5 standard [26]. Briefly, according to ISO 10993-5, LMWHA and LMWHA-Fe_3_O_4_ NPs were immersed in the cultured medium with a concentration of 0.2 g/mL at 37 °C for 24 h. Liquid extracts were collected and added to the culture medium of MCF7 cells with concentrations of 0.1, 0.2 and 0.4 mg/mL, respectively. MCF7 cells cultured with 2% dimethyl sulfoxide (DMSO) and cultured medium alone were used as positive and negative controls, respectively. After co-culturing the cells with the liquid extracts for 24 h, the cell viability was determined using the MTT (3-(4,5-Dimethylthiazol-2-yl)-2,5-diphenyltetrazolium bromide) method. In addition, the absorbance was determined at 570/690 nm wavelengths using a microplate reader (EZ Read 400, Biochrom, Holliston, MA, USA).

### 2.7. Determination of Binding Quantity of LMWHA-Fe_3_O_4_ NPs to MCF7 Cells

MCF7 cells were cultured in 6-well culture dishes with densities of 1 × 10^4^, 5 × 10^4^, 1 × 10^5^ and 5 × 10^5^ cells/mL, respectively. After the cells were attached to the plate, the medium was removed and replaced with a new medium containing 1 mg/mL of LMWHA-Fe_3_O_4_ NPs. After culturing the cells for an additional 12 h, the medium was aspirated and the cells were washed twice with PBS to remove the untargeting LMWHA-Fe_3_O_4_ NPs. An amount of 200 mL 0.05% (*v/v*) Triton X-100 was then added to each dish. After cell disruption by three freeze/thaw cycles, cell lysate from each dish was transferred to 1.5-mL microcentrifuge tubes. The thiocyanate method (see Section 2.4) was then used to measure the binding amount of LMWHA-Fe_3_O_4_ NPs to the MCF7 cells.

### 2.8. Time-of-Flight Secondary Ion Mass Spectrometry Analysis

MCF7 cells were cultured in 6-well culture dishes with a density of 5 × 10^5^ cells/mL. After the cells were attached to the culture plate, the medium was removed and replaced by new media containing 1 mg/mL oleic acid-coated Fe_3_O_4_ NPs, LMWHA and LMWHA-Fe_3_O_4_ NPs, respectively. After co-culturing for 24 h, the media were aspirated and the cells were washed twice with PBS to remove the untargeting materials. The treated cells were fixed by glutaraldehyde treatment. Time-of-flight secondary ion mass spectrometry (TOF-SIMS) (PHI TRIFT IV, ULVAC-PHI, Kanagawa, Japan) was then used to evaluate the targeting status of the LMWHA-Fe_3_O_4_ NPs on the MCF7 cell surface [27]. In performing the TOF-SIMS process, the Bi_3_^+^ primary ion beam (operated at 30 keV) was supplied by a Bi liquid metal ion gun fitted to the instrument. The distributions of the iron ions and phosphocholine fragments in the phospolipid were identified by the *m/z* 56 and *m/z* 86 signals, respectively. In addition, secondary ion images were obtained by scanning the ion beam across the cell surface over an area of 200 × 200 µm.

### 2.9. Statistical Analysis

For the cell viability and iron concentration tests, the mean values and standard deviations of each measurement were recorded. One-way analysis of variance (ANOVA) with Tukey’s post hoc (SPSS Inc., Chicago, IL, USA) tests were then performed to evaluate the differences between the samples. A *p*-value lower than 0.05 was considered to be statistically significant in every case.

## 3. Results and Discussion

### 3.1. Characterization Results for Fe_3_O_4_ NPs

**Figure 1:**
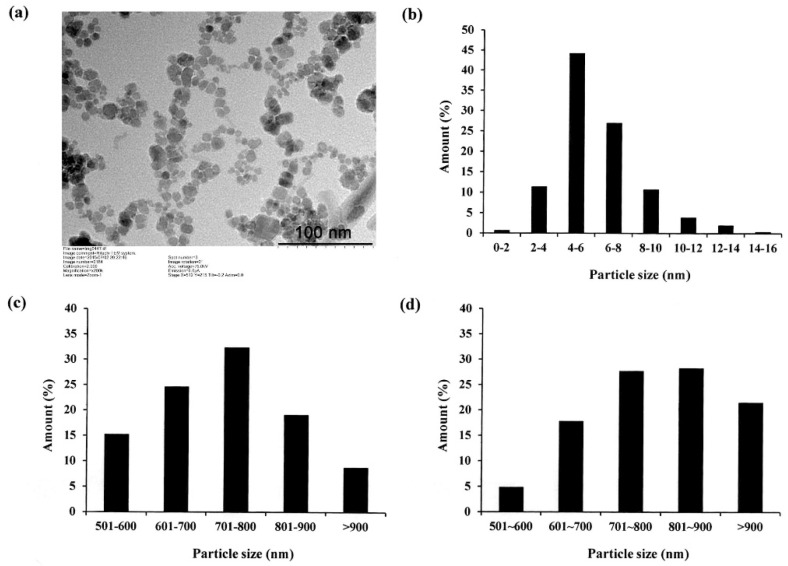
(**a**) Transmission electron microscopy (TEM) image of oleic acid-coated Fe_3_O_4_ NPs and (**b**) particle diameter distribution of nanoparticles; (**c**,**d**) particle diameter distributions of LMWHA and LMWHA-Fe_3_O_4_ NPs, respectively.

In preparing Fe_3_O_4_ NPs, preventing particle aggregation and obtaining a good dispersibility is an important concern. This problem is commonly addressed by coating the surface of the NPs with some form of polymer [7]. The polymer helps the NPs bind to other substances, and hence inhibits their aggregation. However, many polymer coatings may exhibit cytotoxicity, and therefore suppress cell differentiation and may cause cell death and apoptosis [8]. Accordingly, in the present study, the Fe_3_O_4_ NPs were coated with biocompatible oleic acid. The acid not only prevents oxidation reaction, but also suppresses the aggregation of the NPs, and hence reduces their size [28]. Figure 1a presents a TEM image of the oleic acid-coated NPs. It can be seen that the NPs have a spherical shape and are well dispersed in the cultured medium. The results are thus consistent with those presented in a previous report for oleic acid-coated magnetite NPs [29]. As shown in Figure 1b, the NP particle size is distributed mainly (71%) in the range of 4 to 8 nm and is less than 20 nm in every case. 

**Figure 2:**
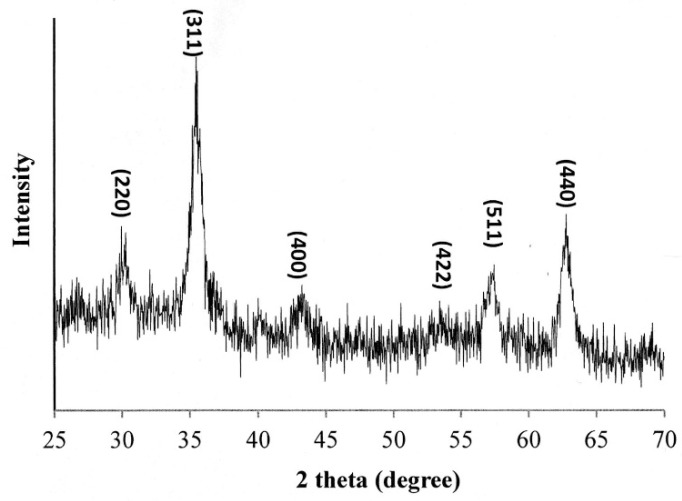
XRD pattern of oleic acid-coated Fe_3_O_4_ NPs.

Figure 2 presents the X-ray diffraction pattern of the oleic acid-coated Fe_3_O_4_ NPs. The sharp diffraction peaks at 2θ = 30.1°, 35.4°, 43.1°, 53.2°, 56.9° and 62.52°, respectively, indicate that the NPs have an inverse spinel structure [30,31]. In other words, the success of the Fe_3_O_4_ NP synthesis process is confirmed.

### 3.2. Characterization Results for LMWHA-Fe_3_O_4_ NPs

The LMWHA prepared by 20 kGy γ-irradiation exposure was found to have a molecular weight of 230 kDa. In general, Fe_3_O_4_ NPs coated with oleic acid can only be dissolved in organic solvents (i.e., not in water). However, HA is extremely hydrophilic and is insoluble in organic solvents. Accordingly, a mixing problem occurs at the interface between the oleic acid-coated F_3_O_4_ NPs and the LMWHA. Previous studies have proposed two methods for overcoming this problem [25]. In the first method, poly (ethylene glycol), poly (ethylene oxide), or poly (vinyl alcohol) is used to modify the surface of the Fe_3_O_4_ NPs. In the second method, the chemical structure of the HA is modified in some way, e.g., the carboxyl group of HA can be used to reduce its hydrophilicity [32]. However, excessive modification may reduce the targeting ability of the HA to the CD44 receptors of the cancer cells. In 2014, Chan et al. reported that the oleic acid coated on Fe_3_O_4_ NPs can be easily replaced by a polymer having more carboxylic acid or phosphate functional groups [32]. Accordingly, in the present study, alkaline solution was used to accelerate the saponification reaction of the oleic acid. Once the saponification reaction is completed and the supernatant was removed, the LMWHA-Fe_3_O_4_ NPs formed.

Figure 1c,d shows the particle size distributions of the neat LMWHA gel and LMWHA-Fe_3_O_4_ NPs, respectively. As shown in Figure 1c, the LMWHA particle size falls mainly in the range of 600–900 nm. Referring to Figure 1d, the number of LMWHA-Fe_3_O_4_ NPs with a size larger than 700 nm (77.4%) is much higher than that of LMWHA particles with a similar size (60.2%). This finding is reasonable since the measured particle size in Figure 1d reflects the total value of HA and many iron oxide NPs [33]. The iron ion content of the LMWHA-Fe_3_O_4_ NPs was evaluated using the thiocyanate method (see Section 2.4). After quantifying the red ferric thiocyanate complex, the iron ion content was determined to be 4.43%.

The stability of the colloidal dispersions of the LMWHA-Fe_3_O_4_ NPs in water was evaluated by means of zeta potential measurements. The zeta potential of oleic acid-coated Fe_3_O_4_ NPs and LMWHA-Fe_3_O_4_ NPs were found to be −45.30 and −43.84 mV, respectively. These values were consistent with the value reported in the literature [34]. In general, the Zeta measurement result indicates that the LMWHA-Fe_3_O_4_ NPs have excellent colloidal stability due to a charge repulsion effect, which inhibits their aggregation in aqueous solutions.

### 3.3. Hysteresis Loop Detection

**Figure 3:**
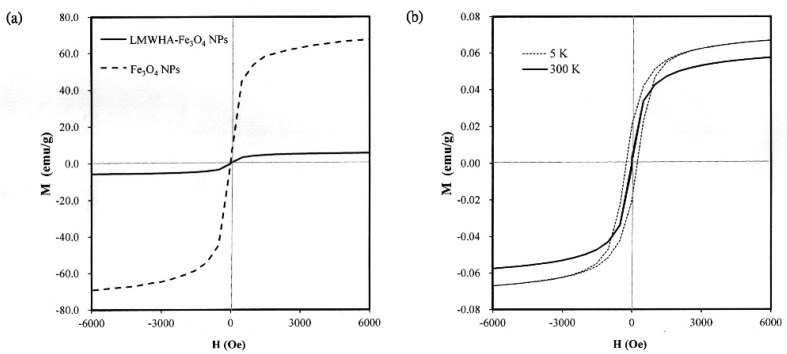
(**a**) Saturation magnetization of acid-coated Fe_3_O_4_ NPs and LMWHA-Fe_3_O_4_ NPs at temperature of 300 K. (**b**) Hysteresis loops of acid-coated Fe_3_O_4_ NPs and LMWHA-Fe_3_O_4_ NPs at 5 K and 300 K.

Previous studies have shown that Fe_3_O_4_ NPs with a diameter lower than 30 nm can pass through a superparamagnetic-ferromagnetic transition and exhibit superparamagnetic behavior [35,36]. As described in Section 3.1, the present Fe_3_O_4_ NPs have a diameter of less than 20 nm (see Figure 1b). Thus, it is reasonable to assume that they may exhibit a superparamagnetic behavior. However, previous studies have neither confirmed nor disproved the existence of such a phenomenon when the nano-particles are used to modify LMWHA. To address this gap, the present study investigated the magnetic properties of the prepared Fe_3_O_4_ NPs and LMWHA-Fe_3_O_4_ NPs using a quantum interference technique. As shown in Figure 3a, the saturation magnetization of the Fe_3_O_4_ NPs was found to exceed 60 emu/g when exposed to a high-strength magnetic field at 300 K. However, the saturation magnetization of the LMWHA-Fe_3_O_4_ NPs was only 5.9 emu/g due to the low concentration (4.43%) of Fe_3_O_4_ NPs in the complex. This finding is consistent with the results of previous studies, which also showed a reduction in the saturation magnetization of Fe_3_O_4_ NPs following modification with nature polymer [37,38]. However, despite the low Fe_3_O_4_ NP concentration ratio, the LMWHA-Fe_3_O_4_ NPs still exhibit a typical superparamagnetic property [36]. That is, a hysteresis loop is not observed at 300 K, but can be seen at 5 K (Figure 3b). Notably, this finding suggests that the LMWHA-Fe_3_O_4_ NPs have significant potential as an MRI contrast agent that can respond to a strong magnetic field [31].

### 3.4. Viscosity Analysis

**Figure 4:**
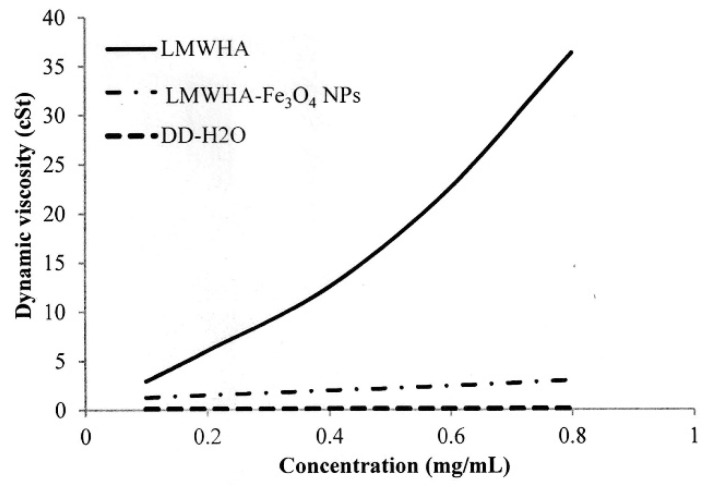
Dynamic viscosities of LMWHA-Fe_3_O_4_ NPs and neat LMWHA with various concentrations.

In practice, the high viscosity of HA limits its application as a blood vessel-injectable material into the human body. Furthermore, even though the viscosity of HA can be reduced by lowering its molecular weight, the efficiency of such an approach depends significantly on the particular method used. For example, while γ-irradiation and enzyme treatment can both reduce the molecular weight of HA, the depolymerization process and breaking site of the HA structure are different in each case. Huang et al. (2019) found that γ-irradiated HA exhibits a Newtonian liquid viscosity behavior due to the collapse of the macromolecular coils during the depolymerization process [17]. Figure 4 shows the measured kinematic viscosities of the present LMWHA and LMWHA-Fe_3_O_4_ NPs. It can be seen that even though an γ-irradiation process was used to fabricate the LMWHA, the dynamic viscosity of the LMWHA is still significantly higher than that of water. However, when the LMWHA is used to modify the Fe_3_O_4_ NPs, the kinematic viscosity decreases to just 1.27–3.00 cSt over the considered concentration range of 0.1–0.8 mg/mL. These values are very close to those of water. In 2012, Fakhari et al. tested the rheological behavior of HA/NP mixtures. Their results also showed that the addition of HA NPs can reduced the viscosity of HA to a value very close to those of water [39]. In other words, modification of the LMWHA by the Fe_3_O_4_ NPs results in a significant reduction in its viscosity and hence improves its potential as a blood vessel-injectable material. Although how NPs can control rheological properties of HA solutions is less well known, the mechanism whereby increasing the relative proportion of NPs in the solution reduces the viscosity of the HA/NP mixtures should be achieved by interrupting HA self-association. This is because the participating sites were occupied, and thus accessibility was reduced [39]. In this regard, it is speculated that the reduction in viscosity showed in Figure 4 is the result of an altered electrostatic attraction between the HA polymer and the Fe_3_O_4_ NPs, which reduces the intermolecular force between the HA and HA molecules.

### 3.5. Thermogravimetric Analysis

**Figure 5:**
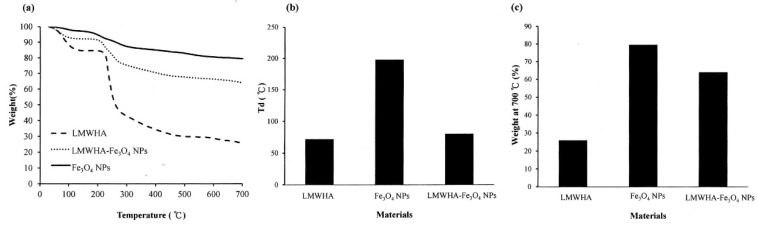
(**a**) Thermogravimetric (TGA) patterns of Fe_3_O_4_ NPs, LMWHA-Fe_3_O_4_ NPs and neat LMWHA. (**b**) Td values and (**c**) residual weights of Fe_3_O_4_ NPs, LMWHA-Fe_3_O_4_ NPs and neat LMWHA.

Previous studies have reported that the addition of Fe_3_O_4_ NPs to polylactide acid improves the thermal stability of the polymer [40]. The TGA analysis results obtained in the present study show that the prepared LMWHA, Fe_3_O_4_ NPs and LMWHA-Fe_3_O_4_ NPs have decomposition temperatures (T_d_) of 71.75 °C, 197.8 °C and 80.1 °C, respectively (see Figure 5a,b). In other words, the addition of the nano-Fe_3_O_4_ particles increases the thermal stability of the LMWHA polymer due to their high T_d_ temperature. In addition, the presence of the Fe_3_O_4_ NPs also increases the residual mass left after the composites have undergone thermogravimetric testing at 700 °C (see Figure 5c). In particular, the char residuals at the end of the TGA runs of the LMWHA, Fe_3_O_4_ NPs and LMWHA-Fe_3_O_4_ NPs are 25.82%, 79.41% and 63.94%, respectively. Notably, the thermal residual weight of the LMWHA-Fe_3_O_4_ NPs obtained in the present study is almost twice that reported in a previous study [25]. It is hence inferred that the Fe_3_O_4_ NPs prepared through the synthesis route proposed in the present study have a higher iron ion content, and are thus more suitable for MRI imaging and/or hyperthermia therapy.

### 3.6. Cytotoxicity Characterization Results

**Figure 6:**
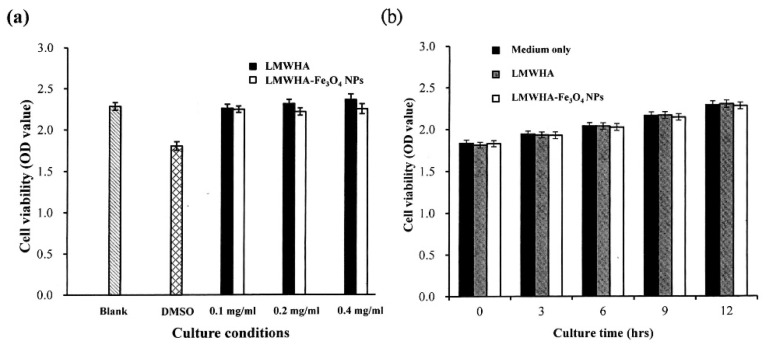
(**a**) Cytotoxicity test results, and (**b**) cell proliferation assay results for MCF7 cells cultured with neat LMWHA and LMWHA-Fe_3_O_4_ NPs.

**Figure 7:**
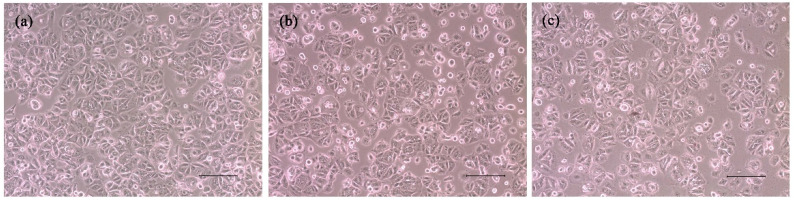
Morphologies of MCF7 cells cultured with (**a**) material-free pure medium, and media with (**b**) neat LMWHA and (**c**) LMWHA-Fe_3_O_4_ NPs. Scale bar denoted 100 μm.

Figure 6 shows the in vitro test results for the cytotoxicity of the prepared LMWHA and LMWHA-Fe_3_O_4_ NPs toward the MCF7 cells. As shown in Figure 6a, the viability of the MCF7 cells cultured with 2% DMSO is reduced by around 20% compared to the blank condition. However, there is no change in cell viability is found under co-culturing with liquid extracts of neat LMWHA and LMWHA-Fe_3_O_4_ NPs. No significant difference is observed in the viabilities of the MCF7 cells co-cultured with the neat LMWHA and LMWHA-Fe_3_O_4_ NPs, respectively, for the considered concentrations of less than 0.4 mg/mL. It was reported that the cytotoxicity of the Fe_3_O_4_ nanoparticles is greatly dependent on the particle size. Xie et al. (2016) investigated the cytotoxic effects Fe_3_O_4_ NPs with different diameters on the human hepatoma cells. Their results indicated that 6 nm Fe_3_O_4_ NPs exhibited negligible cytotoxicity. However, Fe_3_O_4_ NPs with particle size larger than 9 nm may affect cytotoxicity by inducing cellular mitochondrial dysfunction or impairing the integrity of plasma membrane [41]. The particle size of the prepared Fe_3_O_4_ NPs is mainly concentrated between 4 and 6 nm, and this is the reason the Fe_3_O_4_ NPs prepared in this study showed no cytotoxic effects on cells. Furthermore, for both materials, the cell viability increases over the considered 12 h culture period, as shown in Figure 6b. Figure 7 shows that no significant morphological change of the MCF7 cells occurs following co-culturing with liquid extracts of neat LMWHA and LMWHA-Fe_3_O_4_ NPs, respectively. Overall, the results presented in Figure 6 and Figure 7 show that neither neat LMWHA nor the LMWHA-Fe_3_O_4_ NPs exert a cytotoxic effect on the MCF7 cells. Previous studies indicated that γ-irradiation-treated HA significantly showed improvement effect on the viability of fibroblasts [17,42]. However, a comparable improvement in the MCF7 cell viability was not observed (Figure 6b), as shown in previous studies [17,42]. The apparent discrepancy between the two sets of results most likely arises due to the cells were co-cultured with liquid extract rather than with the prepared material directly.

### 3.7. Time-of-Flight Secondary Ion Mass Spectrometry Analysis

**Figure 8:**
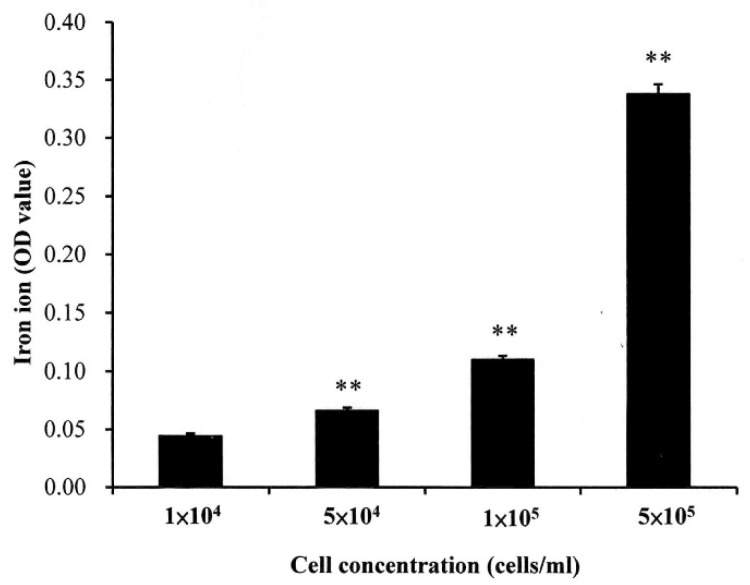
Quantity of iron ions on surface of MCF7 cells with different concentrations. ** *p* < 0.01.

As shown in Figure 4, the dynamic viscosity property alternation of the prepared LMWHA-Fe_3_O_4_ NPs may also affect its targeting ability on the cell surface. However, this phenomenon has not been investigated or discussed previously. The Fe_3_O_4_ NPs targeting on the MCF7 cell surface were extracted to form a solution containing iron ions. The ion concentration was then determined using the thiocyanate colorimetry technique described in Section 2.4. As shown in Figure 8, the extracted quantity of Fe^3+^ ions increased significantly with increasing cell concentration. However, the iron ion amount does not seem to increase proportionally with the increase of cells number. This phenomenon may be due to the HA solved in water was not in a homogeneous status.

It has been reported that TOF-SIMS is a highly sensitive and chemically specific analytical tool for both inorganic and organic subjects [43,44,45]. In the TOF-SIMS process, a high-energy ion beam (referred to as the primary ion beam) is applied to the sample and used to generate ionized molecular fragments from the sample surface. The ionized fragments (referred to as secondary ions) are then collected by a time-of-flight mass analyzer and separated according to their mass-to-charge ratio (*m/z*). The collected data are then used to reconstruct the chemical distribution of the sample surface [46]. For biological applications, TOF-SIMS is an effective technique for analyzing the chemical composition of cellular membranes with a few molecular layers in depth [44] and can identify the chemical change of phospholipid molecules on the surface of single cells or tissues [27,45,46,47,48,49]. Furthermore, TOF-SIMS can be used to analyze whether inorganic material or metal is incorporated in the extracellular matrix and to detect the uptake (or otherwise) of metal iron by human cells [43,45].

**Figure 9:**
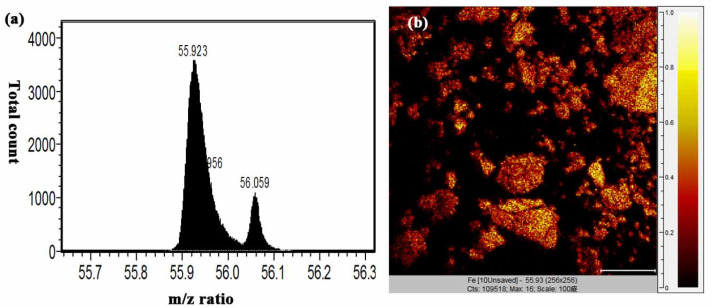
(**a**) *m/z* 56 signal and (**b**) TOF-SIMS image of fabricated Fe_3_O_4_ NPs.

**Figure 10:**
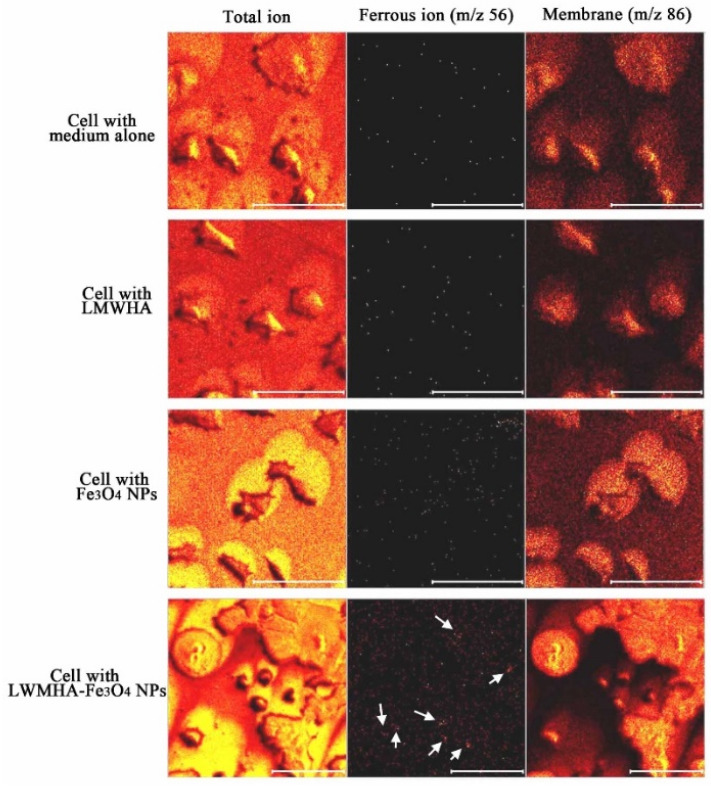
TOF-SIMS images of MCF7 cells cultured with medium alone, LMWHA, Fe_3_O_4_ NPs, and LMWHA-Fe_3_O_4_ NPs. Please note that the *m/z* 56 signal (red dots identified by white arrows) can be found only for cells cultured with LMWHA-Fe_3_O_4_ NPs. Note also that *m/z* 86 images represent cellular membrane.

**Figure 11:**
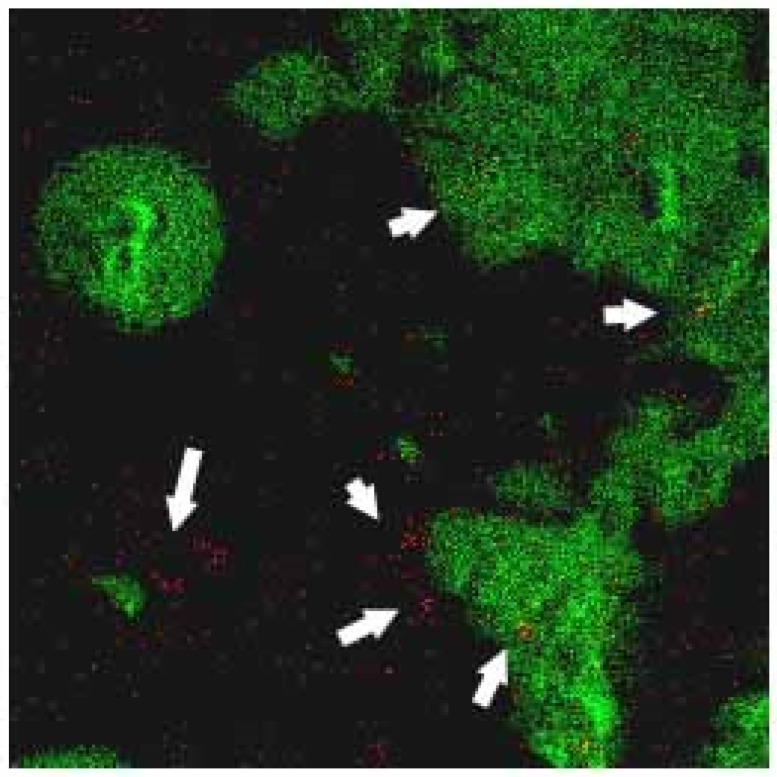
Convolution TOF-SIMS image consisting of *m/z* 56 signal (red image representing iron ion) and *m/z* 86 mass signal (green image representing membrane). Please note that Fe_3_O_4_ NPs (red dots identified by white arrows) are found to target MCF7 cellular surface.

Figure 9a,b shows the TOF-SIMS signal intensity and image, respectively, of the Fe_3_O_4_ NPs (*m/z* 56) prepared in the present study. It is well known that phospholipids are the most abundant molecules on mammalian cellular membranes. The signal at *m/z* 86 represents a fragment (C_5_H_12_N^+^) of phosphocholine; a head group of the phospholipids [43,44]. Thus, in TOF-SIMS analysis, the *m/z* 86 image reflects the chemical composition of membranes [44]. As shown in Figure 10, a high-intensity *m/z* 86 signal exists in the central areas of the present MCF cells, as also described in a previous study [44]. Furthermore, no 56 signal is observed for the MCF7 cells cultured with medium, LMWHA or Fe_3_O_4_ NPs alone. However, for the MCF7 cells cultured with LMWHA-Fe_3_O_4_ NPs, a visible iron ion signal is found. Figure 11 shows the relations between the total ions, *m/z* 56 and *m/z* 86. The convolution image of *m/z* 56 and 86 indicates that the Fe_3_O_4_ NPs exist on the cellular surface. 

Except for TOF-SIMS, scanning (SEM) and transmission (TEM) electron microscopy are techniques that can also provide information regarding the interactions between cells and the prepared LMWHA-Fe_3_O_4_ NPs. However, the sensitivity of the SEM is such that it is hard to observe a single ion. The limitation of this study is that only several layers of atoms can be observed using the TOF-SIMS technique. Thus, whether the prepared Fe_3_O_4_ NPs enter the cells cannot be observed as TEM imaging. However, the detection of biochemical responses due to the NPs enter the cell was not the purpose of this study. Thus, the conclusion of this study would not be changed even without TEM images. Overall, the results present in this study suggest that the prepared LMWHA-Fe_3_O_4_ NPs has significant potential to be developed as an injectable agent for targeting breast cancer tumors in biomedical application.
